# Misdiagnosis of Acute Appendicitis in the Emergency Department: Prevalence, Associated Factors, and Outcomes According to the Patients' Disposition

**DOI:** 10.31486/toj.23.0051

**Published:** 2023

**Authors:** Hila Weinberger, Abdel-Rauf Zeina, Itamar Ashkenazi

**Affiliations:** ^1^ Department of Obstetrics, Gynecology and Reproductive Science, Hillel Yaffe Medical Center, Hadera, Israel; ^2^ Rappaport Faculty of Medicine, Technion-Israel Institute of Technology, Haifa, Israel; ^3^ Radiology Department, Hillel Yaffe Medical Center, Hadera, Israel; ^4^ Department of General Surgery, Rambam Medical Center, Haifa, Israel

**Keywords:** *Abdominal pain*, *appendicitis*, *delayed diagnosis*, *emergency service–hospital*, *missed diagnosis*

## Abstract

**Background:** Although abdominal pain is one of the most common complaints of patients presenting to the emergency department (ED), and acute appendicitis is a leading surgical differential diagnosis of patients presenting with abdominal pain, the diagnosis of acute appendicitis remains challenging. We examined the missed diagnosis rate of acute appendicitis in one ED and evaluated the association between disposition (discharge home or hospitalization in the wrong department) and complicated appendicitis.

**Methods:** We retrospectively evaluated the medical records of patients with acute appendicitis and periappendicular abscess from January 1, 2013, to December 31, 2016.

**Results:** The diagnosis of acute appendicitis was missed in 7.1% of patients (90/1,268) at their first ED encounter: 44 were discharged, and 47 were hospitalized with a wrong diagnosis (1 female patient was both discharged and then hospitalized with an incorrect diagnosis). Compared to the patients who were correctly diagnosed, patients with a missed diagnosis were older (median age 29 years vs 23 years, *P*=0.022), their time between ED first encounter and surgery was longer (median 29.5 hours vs 9.3 hours, *P*<0.001), and their rate of complicated appendicitis was higher (54.4% vs 27.5%, *P*<0.001). Missed females were more commonly hospitalized (26/39), while missed males were more commonly discharged from the ED (31/52) (*P*=0.019). No differences in the time between the first ED encounter and surgery (29.6 hours vs 29.6 hours, *P*=0.29) and the rate of complicated appendicitis (63.8% vs 43.2%, *P*=0.06) were noted between hospitalized patients with a wrong diagnosis and those discharged from the ED. Of the 25 patients with periappendicular abscesses, only 3 could be related to missed diagnoses during their first encounter in the ED.

**Conclusion:** We found that 7.1% of patients were missed during their first encounter in the ED. Hospitalization in departments other than general surgery was not protective against delay in surgery or the development of complicated appendicitis. Periappendicular abscess was attributable to late referral rather than a missed diagnosis in most patients.

## INTRODUCTION

Abdominal pain is one of the most common complaints of patients presenting to the emergency department (ED), and acute appendicitis is a leading surgical differential diagnosis of patients presenting with abdominal pain.^1,2^ The mainstay of diagnosis is clinical suspicion. Diagnosis is based on an appropriate story and an appropriate physical examination with the addition of laboratory tests and directed imaging. Nevertheless, the diagnosis of acute appendicitis remains challenging. Most of the effort during the workup in the ED is aimed at improving the diagnosis of the disease and reducing the rate of unnecessary surgeries for noninflamed appendixes.^3-9^

The importance of timely diagnosis stems from the understanding that the course of appendicitis is time-dependent.^10^ An appendix with simple inflammation may eventually develop necrosis and even perforation. Patients who are not diagnosed on time may develop generalized peritonitis and abscess with a possible increase in morbidity and mortality. Delay in the diagnosis of appendicitis is one of the most common causes of malpractice claims in the United States.^11,12^

In this study, we evaluated the prevalence of missed diagnoses of acute appendicitis during patients’ first encounter in one medical center in Israel. We defined *missed diagnoses* as cases when patients were not referred from the ED to the general surgery department for possible acute appendicitis. Because the impact of disposition has not been evaluated in previous studies,^13^ we stratified patients in this study according to whether they were discharged home from the ED or hospitalized in departments other than the general surgery department with the wrong diagnosis. Our objective was to evaluate the association between wrong disposition and the finding of complicated appendicitis.

## METHODS

### Study Design

This retrospective observational cohort study was approved by the Hillel Yaffe Medical Center Research Ethics Committee, and the need for informed consent was waived (protocols 0130-17-HYMC, 0019-23-HYMC). This study was based on a larger cohort of patients used in 2 previously published studies (see Acknowledgments) examining the association between early imaging, surgery time, and operative findings in patients treated for possible appendicitis. This study differs from the previous 2 studies in purpose and inclusion criteria. The study Patient Time and In-Hospital Delay of Surgery Association With Complicated Appendicitis was registered as ClinicalTrials.gov identifier NCT04689906.

### Study Setting and Population

Included in this study were male and female patients of all ages who were diagnosed with acute appendicitis or periappendicular abscess in a single public hospital in Israel between January 1, 2013, and December 31, 2016. We evaluated all medical records indicating an appendectomy was performed (*International Classification of Diseases, Ninth Revision* [ICD-9] codes 47.0-47.99) or periappendicular abscess was diagnosed (ICD-9 code 540.1). Patients excluded from the study were those who underwent incidental appendectomies, had surgery for indications other than acute appendicitis, and had surgery but had a noninflamed appendix. Patients admitted to the ED with suspicion of acute appendicitis or periappendicular abscess are hospitalized in the general surgery department or its associated pediatric surgery unit. The study group was composed of patients with a final diagnosis of acute appendicitis or periappendicular abscess who were wrongly diagnosed during their first encounter in the ED (ie, missed diagnosis). Patients immediately hospitalized in the general surgery department or its associated pediatric surgery unit served as controls.

### Procedures

We reviewed patient medical records for age, sex, the onset of symptoms (≤24 hours or >24 hours), and operative findings. Patient disposition following the first encounter in the ED was noted and classified as follows: hospitalized in the general surgery department (which includes a pediatric surgery unit), discharged from the ED, or hospitalized in a department other than the general surgery department. Patients hospitalized in the general surgery department and its associated pediatric surgery unit were assumed to have been diagnosed correctly. In contrast, those discharged and those hospitalized in other departments were assumed to have been missed, an assumption that represents the real-world situation from the patient viewpoint.

**Figure 1.**
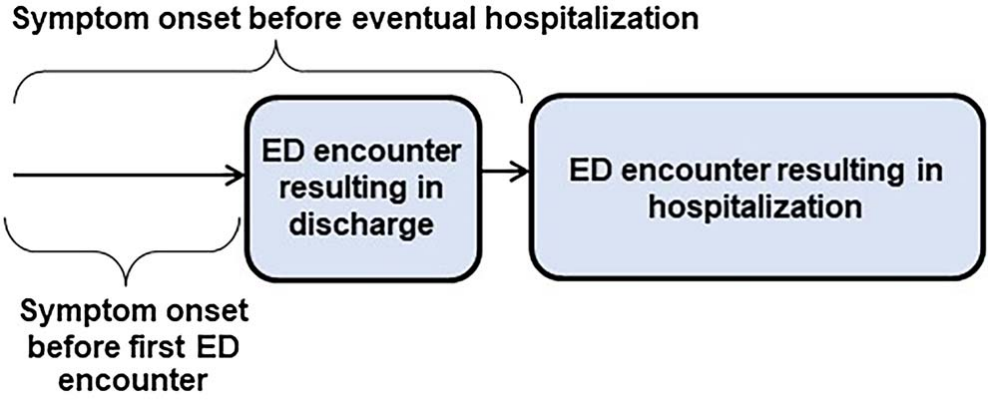
Symptom onset time was noted twice for patients discharged from the emergency department (ED): time before their first encounter in the ED and total time before their eventual hospitalization.

We noted the onset of symptoms twice for patients discharged from the ED. First, we recorded the time of symptom onset before the first encounter in the ED, and then we recorded the total time of symptom onset before the patient's eventual hospitalization (Figure 1). The timing between the first encounter in the ED and surgery was recorded as well.

Operative findings were classified as a noninflamed appendix, noncomplicated appendicitis, or complicated appendicitis. If the operative finding was questionable, the authors reevaluated the pathology report with the pathologists. Patients with noninflamed appendixes were excluded per protocol. Complicated appendicitis was defined as gangrene without perforation, perforation with abscess formation, or free perforation with localized or generalized peritonitis.

### Data Analysis

We calculated the proportion of patients with a missed diagnosis and compared the differences in age between patients who were missed and patients who were identified using the Mann-Whitney test. Differences in sex, symptom onset (≤24 hours or >24 hours), and complicated appendicitis between the 2 groups were assessed using Fisher exact test. Similar statistical tests were used in the comparison of patients discharged home and those hospitalized in departments other than general surgery. Symptom onset was recorded twice. It was first recorded at the first encounter in the ED regardless of whether the patient was eventually hospitalized or discharged from the ED. Symptom onset was reevaluated a second time before eventual hospitalization for patients wrongly discharged from the ED (Figure 1). We compared differences in the time between the first encounter in the ED and surgery between patients who were ED discharged (but eventually hospitalized) and patients who were hospitalized in departments other than general surgery using the Mann-Whitney test. Data were analyzed using dedicated statistical software programs GraphPad InStat version 3.06 for Windows, GraphPad Prism version 6.00 for Windows (GraphPad Software Inc), and SPSS Statistics version 16.0 for Windows (IBM Corporation). *P* values <0.05 were considered significant. Numbers, percentages, and interquartile ranges (IQR) were approximated to the nearest decimal, insignificant *P* values to the nearest hundredth, and significant *P* values and 95% CIs to the nearest thousandth.

## RESULTS

### Patient Population

**Figure 2.**
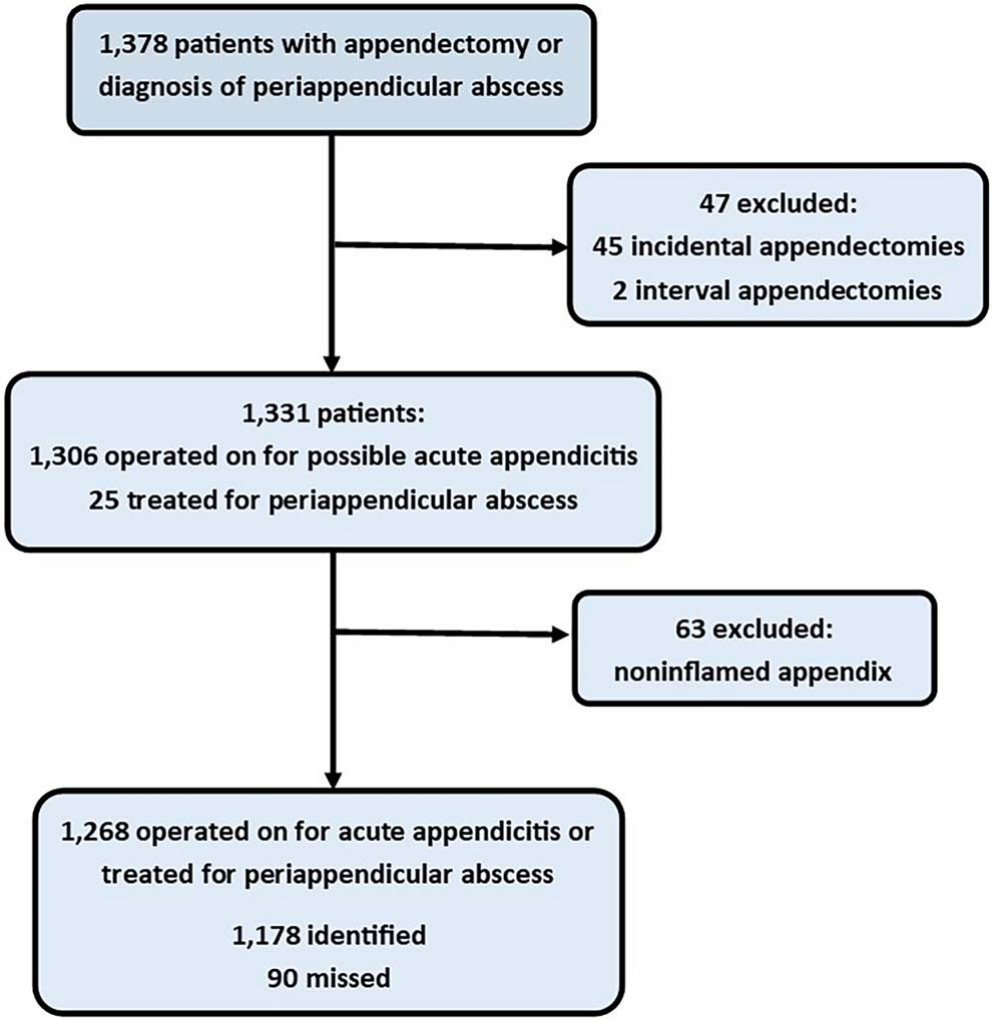
Patient identification and inclusion in the study analysis.

We examined the records of 1,378 patients who underwent appendectomy or were diagnosed with periappendicular abscess. Of these patients, 1,268 were included in this study (Figure 2). The diagnosis of appendicitis was missed in 90 (7.1%) patients during their first encounter in the ED, and they were either discharged home or hospitalized in a department other than general surgery for the wrong diagnosis.

The study population is described in the Table. Age was associated with a missed diagnosis. Delays between ED first encounter and surgery resulted in a higher rate of complicated appendicitis in patients with a missed diagnosis vs patients with an identified diagnosis (54.4% vs 27.5%, *P*<0.001).

Of the 90 patients with a missed diagnosis, 44 (48.9%) were discharged from the ED and 47 (52.2%) were hospitalized in departments other than the general surgery department with the wrong diagnosis (Table). Thirteen patients were hospitalized in pediatrics, 8 were hospitalized in gynecology, and 26 were hospitalized in the internal medicine department. One of these patients is counted twice. The patient was initially discharged from the ED, and upon her return, she was hospitalized in internal medicine with the wrong diagnosis of urinary tract infection. The majority of males (31/52, 59.6%) were discharged, while the majority of females (26/39, 66.7%) were hospitalized in the wrong department. No differences in times between ED first encounter and eventual surgery were noted between patients discharged from the ED and patients hospitalized in the wrong department. The difference in the rate of complicated appendicitis in patients discharged from the ED vs patients hospitalized in the wrong department did not reach significance.

**Table.** Characteristics and Outcomes of the Study Population

	Study Population				Missed Diagnosis Subgroups^b^			
Variable	Missed Diagnosis Group, n=90	Identified Diagnosis Group, n=1,178	Relative Risk (95% CI)	*P* Value	Discharged From ED, n=44	Hospitalized With Wrong Diagnosis, n=47	Relative Risk (95% CI)	*P* Value
Age, years, median [IQR]^a^	29 [18, 43]	23 [14, 39]		0.023	32 [21, 44]	23 [16,42]		0.24
Sex								
Female	38 (42.2)	436 (37.0)	1.22 (0.82, 1.83)	0.37	13 (29.5)	26 (55.3)	0.56 (0.34, 0.92)	0.019
Male	52 (57.8)	742 (63.0)			31 (70.5)	21 (44.7)		
Symptom onset before first ED encounter^a^								
>24 hours	35 (44.3)	584 (51.3)	0.77 (0.50, 1.18)	0.25	11 (31.4)	25 (55.6)	0.56 (0.32, 0.98)	0.042
≤24 hours	44 (55.7)	554 (48.7)			24 (68.6)	20 (44.4)		
Symptom onset before eventual hospitalization^a^								
>24 hours	55 (64.7)	584 (51.3)	1.68 (1.09, 2.58)	0.018	31 (75.6)	25 (55.6)	1.66 (0.95, 2.90)	0.07
≤24 hours	30 (35.3)	554 (48.7)			10 (24.4)	20 (44.4)		
Time between ED first encounter and surgery, hours, median [IQR]^b^^,^^c^	29.5 [21.6, 45.4]	9.3 [6.7, 14.1]		<0.001	29.6 [21.4, 60.5]	29.6 [21.6, 40.7]		0.29
Surgical finding								
Complicated appendicitis	49 (54.4)	324 (27.5)	2.87 (1.93, 4.27)	<0.001	19 (43.2)	30 (63.8)	0.65 (0.42, 1.00)	0.06
Not complicated	41 (45.6)	854 (72.5)			25 (56.8)	17 (36.2)		

^a^Missing data: Age in 2 patients; symptom onset before first ED encounter in 51 patients (40/1,178 in identified group; 11/90 in missed group [9/44 in ED discharged subgroup and 2/47 in hospitalized in the wrong department subgroup; in 90 missed patients there were 91 events, see comment ^b^ below]); symptom onset before eventual hospitalization in 45 patients (40/1,178 in identified group; 5/90 in missed group [3/44 in ED discharged subgroup and 2/47 in hospitalized in the wrong department subgroup]); time between ED first encounter and surgery in 1 patient who refused surgery and following interval appendectomy had evidence of uncomplicated appendicitis.

^b^One patient who was discharged from the ED and upon return was hospitalized with the wrong diagnosis in the wrong department was counted twice for a total of 91 events in 90 patients.

^c^Patients with diagnosis of periappendicular abscess are excluded.

Note: Data are presented as n (%) unless otherwise indicated.

ED, emergency department.

### Periappendicular Abscess

**Figure 3.**
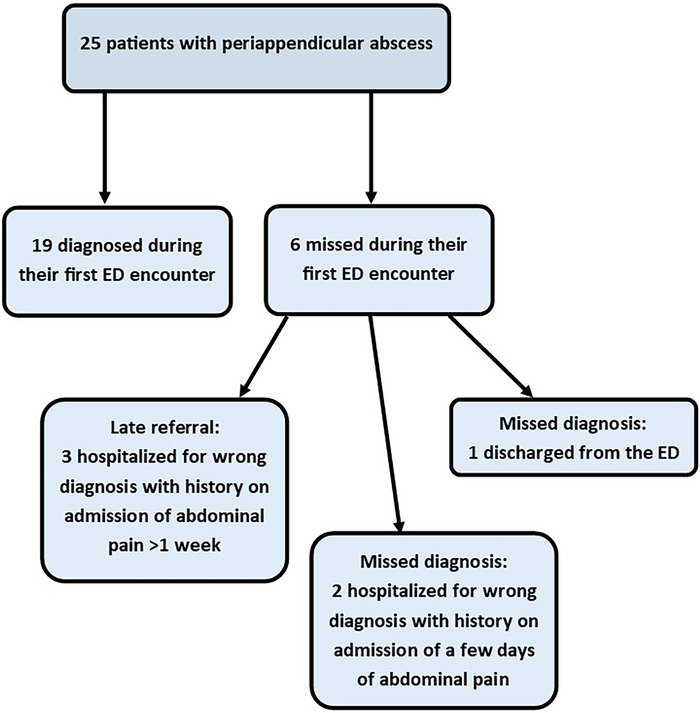
**Late referral vs missed diagnosis in patients with periappendicular abscess.** ED, emergency department.

Twenty-five patients were diagnosed with periappendicular abscess (Figure 3). Of these patients, 19 (76.0%) were diagnosed upon their first encounter in the ED, and 6 (24.0%) were missed. One of the 6 missed patients was discharged from the ED 2 weeks before his final admission with abdominal pain. Four of the other 5 patients were hospitalized in internal medicine, and 1 was hospitalized in pediatrics. In 3 of 5 patients hospitalized in a department other than the general surgery department, symptom onset before admission was >1 week. In the 2 other patients, symptom onset was only several days long. Computed tomography performed 2 and 3 days following admission in these 2 patients revealed the diagnosis of periappendicular abscess.

## DISCUSSION

The primary purpose of this study was to determine the prevalence of patients with acute appendicitis or periappendicular abscess who were not diagnosed during their first encounter in the ED. In addition, we wanted to determine the characteristics of these patients and the consequences of the delay in diagnosis. In the present study, the correct diagnosis was not suspected in 7.1% of the patients. Although this percentage represents a minority of the patients, acute appendicitis is a common disease, and the absolute number of patients with incorrect diagnoses initially is substantial.

Mahajan et al reported a 6.0% missed appendicitis rate in adults and a 4.4% missed appendicitis rate in children.^13^ The Mahajan et al study, based upon 187,461 insurance claims, identified the following possible risk factors for missed appendicitis: female sex, abdominal pain associated with constipation, and the presence of 2 or more comorbidities. Mahajan et al defined missed appendicitis as patients not diagnosed with appendicitis the day they were referred to the ED. No differentiation was done between patients discharged and those admitted to a department other than the general surgery department with a wrong diagnosis.^13^

Older age may affect the presenting symptoms and signs of acute appendicitis.^14^ The symptoms may vary in intensity, and peritoneal irritation signs may not be as clearly elicited during the physical examination as in younger patients. Furthermore, the differential diagnosis increases with age. Patients in this study who were missed were older. We expected that older age would also lead to a higher rate of hospitalization in a department other than general surgery rather than discharge. However, this expectation was not true. The median age of patients discharged was 9 years older than the median age of the patients who were hospitalized, but the difference did not reach statistical significance. To better define why older patients tend to be discharged more than younger patients, further studies of patients wrongly discharged should evaluate age against the sign and symptom intensity, laboratory tests, and presumed diagnoses.

Similar to other reports on acute appendicitis,^2^ most of the patients in our study with acute appendicitis were male. However, sex was not a differentiator between those identified and those missed. This finding was unexpected because the differential diagnosis in females includes gynecologic pathologies. Urinary tract inflammation, which may manifest as lower abdominal pain, is also more common in females than in males.^15^

Although the rate of undiagnosed appendicitis was relatively similar between males and females, the disposition following the wrong diagnosis was dissimilar between the 2 sexes. Most of the missed males were discharged, while most of the missed females were admitted to a department other than general surgery with a wrong diagnosis. This outcome was expected because of the differential diagnosis of gynecologic and urinary pathologies that are more common in female patients.

The natural course of appendicitis is time-dependent. Many patients not diagnosed in a timely way develop appendiceal gangrene and perforation.^10^ The results of our study show that complicated appendicitis increased from 27.5% (324/1,178) in patients identified during their first encounter in the ED to 43.2% (19/44) of patients discharged from the ED and 63.8% (30/47) of patients admitted to departments other than general surgery.

One would expect that hospitalized patients would soon be diagnosed as having acute appendicitis rather than receiving an alternative diagnosis. The findings of this study suggest otherwise. Hospitalization with the wrong diagnosis did not confer any advantage compared to ED discharge. Times between ED first encounter and surgery were the same in the 2 missed diagnosis subgroups, and the difference observed for complicated appendicitis did not reach significance.

Included in this study were 25 patients in whom periappendicular abscess was diagnosed. Formation of a periappendicular abscess is time-dependent and therefore related to late diagnosis.^10^ In this study, the majority of the patients with periappendicular abscess presented late. A review of the medical records of the 6 patients whose diagnosis was missed during their first encounter in the ED reveals that in 3 patients, the time of symptom onset at this first encounter was 1 week or longer. Thus, we concluded that in all except 3 patients (22/25), periappendicular abscess resulted from late referral rather than a missed diagnosis in the ED.

### Limitations

The major limitation of this study is its retrospective nature. Included in this study were patients who were eventually diagnosed with acute appendicitis or periappendicular abscess. Because we analyzed records at a single center, the patient population does not include patients who may have been discharged from the ED but may have been later diagnosed with acute appendicitis at another hospital.

Many reasons may lead to misdiagnosis of acute appendicitis in the ED, including patient-related (age, sex, and sign and symptom intensity) and physician-related (expertise) variables. This retrospective study is limited in its possibilities to assess the relative contribution of different reasons that may have led to misdiagnosis. A prospective study is needed to ensure data are present to the extent that will not impair analysis. The availability of imaging for evaluating patients in the ED was not a limitation of our study.

We used disposition to define the patients in whom acute appendicitis or periappendicular abscess were missed. We did not analyze the presumed differential diagnosis of these patients, whether they were discharged from the ED or hospitalized in a department other than general surgery. Neither did we analyze whether the diagnosis of acute appendicitis was immediately made in all the patients hospitalized in the general surgery department or its associated pediatric surgery unit. Still, the institution's policy is that all patients with presumed acute appendicitis and periappendicular abscess are hospitalized in the general surgery department. Furthermore, as indicated by the median times and IQR, 75% of those considered identified were operated on within 14.1 hours from registration in the ED.

## CONCLUSION

Few studies have examined the reasons for the delayed diagnosis of acute appendicitis following evaluation in the ED. We are unaware of any studies that assessed the outcomes of patients wrongly diagnosed but still hospitalized in departments other than general surgery. The results of our research show that hospitalization in departments other than general surgery for the wrong diagnosis does not confer protection to these patients. Rather, although these patients are under medical supervision, their delay until surgery following the correct diagnosis of acute appendicitis was the same as those who were wrongly discharged home and returned to the ED for reevaluation of their abdominal pain. Although the rates of correct diagnosis during the first encounter could be improved, we should assume that some patients will always remain undiagnosed. Similar to our practice of warning discharged patients to return to the ED if the pain does not resolve or worsens, we should warn our staff to remain vigilant and reevaluate their working diagnosis in patients whose differential diagnosis may include acute appendicitis.

## Notes

*This article meets the Accreditation Council for Graduate Medical Education and the American Board of Medical Specialties Maintenance of Certification competencies for Patient Care and Medical Knowledge.*
